# Concurrency revisited: increasing and compelling epidemiological evidence

**DOI:** 10.1186/1758-2652-14-33

**Published:** 2011-06-20

**Authors:** Timothy L Mah, James D Shelton

**Affiliations:** 1 Bureau for Global Health, United States Agency for International Development, Washington, DC, USA

## Abstract

Multiple sexual partnerships must necessarily lie at the root of a sexually transmitted epidemic. However, that overlapping or concurrent partnerships have played a pivotal role in the generalized epidemics of sub-Saharan Africa has been challenged. Much of the original proposition that concurrent partnerships play such a role focused on modelling, self-reported sexual behaviour data and ethnographic data. While each of these has definite merit, each also has had methodological limitations. Actually, more recent cross-national sexual behaviour data and improved modelling have strengthened these lines of evidence. However, heretofore the epidemiologic evidence has not been systematically brought to bear. Though assessing the epidemiologic evidence regarding concurrency has its challenges, a careful examination, especially of those studies that have assessed HIV incidence, clearly indicates a key role for concurrency.

Such evidence includes: 1) the early and dramatic rise of HIV infection in generalized epidemics that can only arise from transmission through rapid sequential acute infections and thereby concurrency; 2) clear evidence from incidence studies that a major portion of transmission in the population occurs via concurrency both for concordant negative and discordant couples; 3) elevation in risk associated with partner's multiple partnering; 4) declines in HIV associated with declines in concurrency; 5) bursts and clustering of incident infections that indicate concurrency and acute infection play a key role in the propagation of epidemics; and 6) a lack of other plausible explanations, including serial monogamy and non-sexual transmission. While other factors, such as sexually transmitted infections, other infectious diseases, biological factors and HIV sub-type, likely play a role in enhancing transmission, it appears most plausible that these would amplify the role of concurrency rather than alter it. Additionally, critics of concurrency have not proposed plausible alternative explanations for why the explosive generalized epidemics occurred. Specific behaviour change messaging bringing the concepts of multiple partnering and concurrency together appears salient and valid in promoting safer individual behaviour and positive social norms.

## Introduction

Why did generalized HIV epidemics burst upon the scene and persist at high levels only in some 14 countries in eastern and southern Africa? Just as the biology of HIV infection is quite complex, incompletely understood and continues to unfold, the very complex dynamic epidemiology of generalized epidemics remains a formidable challenge. Yet insights continue to emerge. To succeed fully with HIV prevention, as well as other programme efforts, we must continue to improve our understanding of the dynamic of transmission in these generalized epidemics, including why they have only occurred in such few countries.

From the outset, it was recognized that transmission was occurring rapidly in these epidemics and one concept that emerged fairly early was sexual partner concurrency, or overlap of sexual partnerships [1,2]. Clearly, it is impossible to have a sexually transmitted epidemic of any sort if individuals have zero or one lifetime partner; multiple sexual partnering has to play a key role. But multiple partnering comes in a wide variety of shapes and sizes, including whether they occur in serial or concurrent patterns. Conceptually, concurrency or overlap allows for efficient transmission through populations [3]. Moreover, once it was recognized that infectiousness was much higher during the acute phase or first few months of infection, it became intuitively appealing that a network of concurrent sexual partnerships allowed for rapid dissemination of infection through sequential acute infection, rather like a chain reaction.

Still, some have questioned whether concurrency in fact plays a key role in generalized epidemics in eastern and southern Africa, over and above simple multiple partnering [3-9]. In our view, these differences arise in large part because of the complexity of the epidemics and the major limitations of some of the tools at hand to assess concurrency. Nevertheless, in view of more recent evidence that has emerged, we believe that the evidence for a key role is now overwhelmingly compelling and that alternative explanations are not sufficient to explain the generalized epidemics of sub-Saharan Africa [10-12]. Notably, while non-sexual transmission (i.e., iatrogenic or parenteral) likely contributes some new infections in generalized epidemics, empirical evidence continues to mount that non-sexual transmission is not the major contributor of new infections in these epidemics [13-17].

## Discussion

### What do we mean by concurrency and what are its implications?

Consistent with the UNAIDS definition, we take concurrency to include any kind of overlap of sexual partnering during a period of time [18]. That would include long-term overlap, such as formal polygamy or quasi-polygamy. It would also include individuals with a long-term partner with sporadic or isolated sex with another partner. It could also include very sporadic sex that overlaps, such as in the case of migrants who have partners in multiple locations. In short, it is essentially any multiple partnering other than serial monogamy. Clearly, there are numerous overlapping partner patterns, each with their own associated risks.

One of the key implications of concurrency is that, in theory, the risk of the person who has the multiple partners (concurrently) is not elevated over and above the risk of having multiple partners alone [19]. Rather, the person with concurrent partners serves as a potential conduit between his or her other partners and thereby puts them at higher risk. With serial monogamy, the person with multiple partners does not serve as such a conduit because a second sexual partnership only starts when a first one has ended. To explain this, Morris uses the example of "an initially concordant negative couple, where one of the partners forms a concurrent partnership. The monogamous partner is now exposed to the possibility of disease transmission, not by his/her own behavior, but by the partner's concurrency" [7].

### Limitations of available evidence

Part of the difficulty in interpreting concurrency is the lack of comparable data, the complexity of sexual partnerships and networks, and the somewhat indirect way in which concurrency is believed to operate. Thus, in examining the evidence for concurrency and other sexual behaviours, several important limitations should be noted.

#### HIV incidence

Though essential to assess the impacts of prevention programmes, HIV incidence is seldom measured, primarily because of current methodological limitations, though it remains *the *crucial impact measure of prevention success [20,21]. HIV prevalence is a poor substitute since it reflects cumulative prior risk and exposure often over many years.

#### Challenges with self-reported sexual behaviour

Accurate measurement of sexual behaviours is difficult and fraught with challenges [22,23]. Self-reported data are often variably affected depending on methods used for data collection. Fixed interview surveys are particularly prone to under-reporting of multiple partners, especially among women [24,25].

#### Lack of comparability of cross-national data on sexual behaviour and on concurrency

If concurrency is to be invoked as an important part of the explanation for why generalized and hyperendemic epidemics have occurred in eastern and southern Africa, it calls for evidence that sexual behaviour patterning and networking is different there. However, strictly comparable behavioural data on concurrency are just not available on a wide cross-national basis.

#### Inconsistent definitions

Only recently has a standardized definition of concurrency been proposed, i.e., overlapping sexual partnerships in which sexual intercourse with one partner occurs between two acts of intercourse with another partner, though numerous indicators exist and continue to be used to operationalize this definition [18]. Current literature and behavioural data use indicators to measure various aspects of concurrency, such as point prevalence, cumulative prevalence, duration of overlap, coital frequency and relational context, making cross-country or cross-population comparisons difficult.

#### Coital frequency

HIV transmission depends on exposure to the virus, which is in turn dependent on coital frequency. However, coital frequency is not often measured on behavioural surveys. Without this key variable, assessing risk from any other factor is difficult and diluted.

#### Partners' behaviours

As we have described, for the individual, partner(s)'s concurrency behaviour is key to that individual's risk. In most behavioural surveys, partners' behaviours (e.g., numbers of their partners) are seldom assessed. Measures of partners' behaviours are also likely affected by reporting bias [26].

#### Difficulty of modelling

Modelling of these complex epidemics is extremely difficult. Even current models, which are vastly improved and provide insights on the generalized epidemics, are still not sufficiently adequate to simulate fully these epidemics. Thus it is easy to criticize modelling, particularly earlier, less sophisticated approaches.

#### Value of ethnographic data

Qualitative and ethnographic data that aim to describe sexual behaviours are at their core context-specific. Thus, while regional ethnography does give the impression that concurrency is more common where generalized epidemics have occurred, it remains open to question.

### New lines of evidence

Criticisms of concurrency have largely focused on: 1) the rudimentary assumptions in early modelling on concurrency; 2) limited ability to discern differences in concurrency patterns in eastern and southern Africa from other parts of the world; 3) weak evidence of concurrency within sexual behaviour data, such as Demographic and Health Surveys (DHS), which suffer from many of the important limitations discussed earlier, including reliance on HIV prevalence and poor/limited data on sexual behaviours; and 4) the lack of a common definition of concurrency [4,5,9]. We do find support for a key role of concurrency within previous modelling, ethnography and analysis of DHS, as well as more recent modelling and survey evidence [3,6-8,27]. However, here we focus on additional compelling evidence, especially epidemiologic evidence that has largely not been part of the discussion.

#### Early and dramatic rise in incidence

By all accounts, the origins of essentially all of the generalized epidemics involved extremely rapid and explosive increases in incidence [28]. The early and dramatic rise in incidence can only be the result of increased levels of exposure between multiple persons during the acute infection phase and thereby concurrency. It is recognized that infectiousness is markedly higher in the first few acute-phase months of infection, followed by extremely low infectiousness of less than one in 1000 acts of intercourse during the subsequent long chronic phase. Infectiousness then begins to increase in the final symptomatic phase many years later [29]. While infectivity during the chronic phase may occasionally increase when co-infected with other sexually transmitted infections, it is unlikely that these sporadic spikes would be sufficient to explain population-level sustained HIV transmission.

Accordingly, at the early stage of these epidemics, acute infections had to play a crucial role because very few people would be in the final phase, and infectivity is so low in the chronic phase. And for acute infection to play such a role over short periods of time requires substantial overlapping partnerships or concurrency. True, these epidemics have all matured and transmission from individuals in the later phases of HIV has clearly increased. But it appears inescapable that this crucial interacting mechanism of synergy between acute infection and concurrency persists to an important extent in the generalized epidemics. Moreover, concurrency can still play an important role in transmission via persons in the two later stages of infection.

#### Evidence of concurrency in incidence studies involving couples

Among the few studies that have measured incidence, a number have found a surprisingly high proportion of new infections to occur in a partnership where neither partner was previously infected. For example, in the Mwanza, Tanzania, cohort, Hugonnet *et al *found that 18 seroconversions (67%) occurred in concordant negative couples "presumably as a result of extramarital exposure" [30]. In the Masaka, Uganda, cohort, Carpenter *et al *report that 25 of 59 (42%) sero-conversions occurred in adults with HIV-negative spouses, while 34 occurred in adults with HIV-positive spouses [31]. In a nationally representative study in Uganda, Mermin *et al *report that among 74 married participants with recent infection and whose spouses were also tested for HIV, 36 (49%) had spouses who were not infected with HIV [32] and an additional 13% were instances where both partners become recently infected. In the Rakai, Uganda, cohort among identifiable married couples, more new infections occurred to a partner in a sero-concordant negative partnership (23%) than to the negative partner in a discordant partnership (18%) [33]. Thus, all four of these studies implicate a very active role of concurrency in propagating the HIV epidemic.

Moreover, careful analysis of new infections, even within discordant couples, finds high rates of infection from outside the primary partnership and thus compelling evidence for concurrency as a source of new infections. For example, Celum *et al *found that 38 of 132 sero-conversions (29%) within discordant couples were determined to not be genetically linked to the enrolled primary partner [34]. The researchers state that this "...probably reflect HIV-1 infection from a person other than the study partner". Similarly, genetic analysis from Zambia and Rwanda among discordant couples found that about a quarter of infections were from an outside partner [35].

#### Elevated risk associated with partner's multiple partnering

Various studies have found that belief that a partner has more than one partner is associated with increased incidence and/or prevalence. To best understand the relationship between concurrency and HIV acquisition, the behaviours of partners must be measured. Notwithstanding the limitations of one partner's knowledge of the other's sexual activity, several studies have found that HIV incidence and/or prevalence is highly correlated with having partners who have or are thought to have other partners.

A study in Uganda found that incident HIV infection was highly correlated with coital frequency with a person who they knew or suspected to have other partners (adjusted RR 6.3; 95% CI 1.73-21.1) [36]. A study among pregnant women in Tanzania found that the strongest correlate of prevalent HIV infection was reporting a partner who "has women outside the relationship" (adjusted OR 15.11; 95% CI 8.39-27.20) [37]. Notably, in these studies, HIV-infected or seroconverting individuals reported their beliefs about partner behaviour before HIV status or seroconversion was assessed and thus were not subject to differential recall bias. Another study in Tanzania found that among monogamous women presenting for HIV counselling and testing, reporting a partner who has other partners was significantly correlated with HIV seropositivity; the correlation was not significant among men [38]. An analysis of the 2004-05 Uganda DHS found that the proportion of couples where one or both partners were HIV positive increased from 2.0% among lifetime mutually faithful partners to 11.5% among not mutually faithful couples [39]. And a recent long-term cohort study of women in Zimbabwe followed from late pregnancy found statistically significant higher HIV incidence among women who knew that their partners had other partners [40].

#### Decline in HIV associated with declines in multiple partnerships and concurrency

A number of studies have found declines in HIV incidence associated with multiple partnering without assessing concurrency *per se*, reinforcing the crucial role of some kind of multiple partnering in these epidemics [41,42]. However, investigators in the large Manicaland cohort in eastern Zimbabwe measured point prevalent concurrency. Their study found a decline in reported concurrency of about 41% and a delay in sexual onset for both sexes over an approximate three-year period beginning in the late 1990s, without an appreciable change in condom use. The decline was coincident with significant reductions in HIV prevalence, especially in younger age groups [43,44].

#### "Bursts" and clustering of infection

Another interesting area of research, which provides insight into the role of concurrency and its interaction with acute infection, is the clustering of infections. A geo-spatial study in South Africa found localized clustering of prevalent HIV infection along the national road in one district [45]. Given the relatively mature nature of the South African epidemic, one would expect a more even geographic spread of infection, the lack of which suggests, among other things, that bursts of infections resulting from acute infection and concurrency or rapid serial partnering play a critical role in onward transmission.

Similarly, phylodynamic studies indicate clustering transmission of HIV. The ability to genetically trace HIV infections serves as a useful tool for understanding how HIV is transmitted in a population. A phylogenetic study among 2126 men who have sex with men (MSM) revealed several insights into that population, including: 1) 25% of individuals were infected with HIV genetically linked to 10 or more individuals; and 2) 25% of the clustered transmissions occurred within six months of infection. Together, these data indicate that transmission was episodic, with acute infection and concurrency (or rapid serial partnering) likely playing a key role [46,47]. A study among heterosexuals in the UK using similar methods found that HIV transmission was clustered, but on average in smaller groups compared with MSM, and that HIV was transmitted with slower dynamics than among MSM [48]. A phylogenetic study in Quebec found that nearly half of primary HIV infections were clustered, again pointing to the key role of acute infection and concurrency or rapid serial partnering [49].

#### Lack of other plausible explanations

Critics of concurrency have had some valid criticism, but they have failed to provide any adequate alternative explanation for why these explosive epidemics occurred. It appears quite clear that in the sexual epidemics, multiple partnering of some kind has to play a vital role. Excluding concurrent partnerships leaves only serial monogamy as an alternative. And it is clear, among other things from modelling, that serial monogamy, even under rather extreme assumptions of partner change, cannot account for these epidemics [50,51]. In contrast, concurrency provides a highly plausible explanation supported by compelling evidence.

### Epidemiologic evidence seemingly not supporting a role of concurrency and HIV incidence

Not all of the epidemiologic data are supportive. As mentioned previously, a study in Tanzania found that among men, prevalent HIV infection was not correlated with reporting a partner who has other partners, though it was among women [38]. A cohort study in South Africa reported higher HIV incidence among women who reported "concurrency" of their own; however, this may represent risk from multiple partners, since partner's concurrency should further increase risk.[52]. A brief description in a follow-up letter indicates little difference in incidence among women suspecting that their partner definitely or probably was having sex with someone else and those not - a surprisingly high 69.6% versus 66.1% [53]. However, the authors acknowledge the limitations of this measure of concurrency. Furthermore, a full analysis is not presented, and a specific analysis needed is the incidence among women who themselves report only one partner.

### Other possible explanatory factors

In addition to sexual behaviour, it is important to consider other factors that might help explain why HIV emerged so robustly in eastern and southern Africa. One of these is other sexually transmitted infections (STIs). Certainly, biologic evidence of both increased infectiousness and susceptibility, as well as some epidemiologic evidence, indicates that particularly ulcerative STIs play some role [54]. However, the preponderance of randomized trials have failed to show an impact of STI treatment at both the individual and community level on HIV transmission [55]. Of course, these other STIs are also profoundly influenced by sexual behaviour and have their own literature implicating a crucial role of concurrency in their transmission [56,57]. Thus it appears that STIs in some instances may heighten transmission, but do not appear to substantially alter the overall dynamic, including a key role of concurrency.

Likewise, there is evidence that certain other infectious diseases, notably malaria and schistosomiasis, may impact HIV sexual transmission [58,59]. However, much of the epidemiology runs counter to a pivotal role for these other infections. For example, HIV rates are highest in southern African countries, such as South Africa, Botswana, Zimbabwe, Namibia, Swaziland and Lesotho, which tend to have lower rates of infectious disease (e.g., malaria, schistosomiasis or intestinal worms) compared with lower HIV burden countries, such as in the regions of west and central Africa [60]. Also, HIV has been positively associated with household wealth, while schistosomiasis and other infectious diseases have been negatively associated with wealth, thereby arguing against a central role for infectious diseases in HIV transmission [61-64]. Thus, as with STIs, it appears that some other infectious disease may amplify sexual transmission to some extent, but do not change the overall sexual pattern dynamic at the population level.

Some evidence also suggests that sub-type C, the HIV sub-type that is most common in southern Africa, may be more aggressive than the other sub-types. For example, a recent study of a cohort in Botswana infected with sub-type C found a high level of viral load for a somewhat longer period of time among about one-third of the newly infected [65]. Actually, such a change might indicate an even more important role of sequential acute infections with sub-type C because of the longer window of acute infection. It might help explain why prevalence is highest in southern Africa, but still implicates the key role for the deadly synergy between acute infection and concurrency. In addition, generalized epidemics have also emerged where sub-types A and D predominate, such as in parts of eastern Africa, though differential sub-type transmission efficiency exists [66]. Lastly, it is conceivable that other factors, such as population susceptibility or sexual practices like dry sex or rough sex, might also play a role in enhancing transmission [67].

Notably, modelling strongly suggests a "take off" or "tipping point" where concurrency and acute infection interact synergistically to dramatically increase transmission rates. Rather than change the overall transmission dynamic of these epidemics, it appears most plausible that these various possible enhancers contribute to that synergy and actually play an amplifying role for concurrency.

### Possible major role of sex work

Another conceivable possibility is that high levels of sex work explain the generalized epidemics. To some extent, sex work could be considered serial monogamy of an extreme form, if sexual contacts with the same individual are not repeated. In situations where sex work recurs with the same client or if a sex worker has a regular partner in addition to clients, the criteria for concurrency would be met. However, data suggest that sex work in a formal sense is relatively uncommon in east and southern Africa. For example, in the most recent DHS in Zimbabwe, Zambia, Uganda, Swaziland and Namibia, less than 2% of men reported sex with a sex worker in the previous year [68]. In South Africa, "one-night encounters", which likely include more than sex work, were uncommon (3%) compared even with other casual partnerships [69]. Even acknowledging under-reporting of sex work, it is unlikely that sufficient proportions of men and women would be engaged in sex work to account for the transmission dynamics that we see in the generalized epidemics.

### Improved modelling still finds a key role for concurrency

It is true, as critics have pointed out, that early modelling of these very complicated epidemics used some unrealistic assumptions [5,9]. However, newer, improved models continue to find a key role for concurrency. For example, Eaton *et al *replicated and extended an earlier model by Morris and Kretzschmar to include the critical three stages of infection [70]. The modellers found not only a key role for concurrency, but a particularly crucial role for the interaction of concurrency with acute infection. A model by Goodreau *et al *similarly suggests that acute infection is strongly moderated by concurrency and that when using empirical data from Zimbabwe, epidemic potential cannot be achieved without both concurrency and acute infection [71].

### Newer sexual survey analysis

Recognizing the diversity and difficulty of comparing sexual behaviour survey data across regions, Morris *et al *have undertaken a recent analysis of reported sexual behaviour among men in three fairly similar national surveys, all from the early 1990s, from Uganda, Thailand and the US [27]. Using cumulative concurrency, the three countries appeared similar. However, they found substantially higher point prevalence concurrency in Uganda with longer duration and considerably greater coital frequency. This analysis does support the qualitative evidence indicating that concurrency patterning in Africa is considerably higher risk than in the other regions. A similar study in the US found that blacks and Hispanics reported higher concurrency prevalence, longer duration and greater coital exposure than whites; using modelling, the study was able to show a 2.6-fold racial disparity in epidemic potential between the populations [72].

### Why not west and central Africa?

If indeed multiple partnering and concurrency are important for the generalized epidemics, why did they not occur in west Africa as well, where limited evidence suggests sexual patterns are not dissimilar [73]? The obvious explanation is that where male circumcision is extremely common, as it is in nearly all of west and central Africa, it provides a level of protection at the population level that resists widespread HIV propagation [74]. One plausible concept is that the 60%-plus protection that male circumcision provides on the individual level is enough to slow down transmission, particularly in the acute phase, such that the tipping point of explosive transmission through acute infection and concurrent sexual networks is not reached.

### What difference does "multiple" versus "concurrent" partners make for programmes?

There appears to be no question that multiple partnering in some way is crucial for the generalized epidemics. So why delve into this issue of concurrency at all in programmes since there may be some question about it, and one's own concurrency theoretically does not add to one's own risk? First, these are closely related concepts that both need to be addressed. But for behaviour change interventions to work, they have to be salient and effective in reaching people with knowledge and actionable change that will help them decrease their risk. Specific concurrency messages add understanding, salience and contextual specificity that transcend messaging purely about reducing partners. They appear both to heighten individual risk perception and promote more positive social norms.

Such message examples include: the idea that there is a sexual network "out there" with actively infected people, that it is risky, and that you need to protect yourself by minimizing exposure to it; you need to be concerned not only about your own behaviour, but also that of your partner(s); risk of multiple partnering not only applies to people with many partners, but also to someone who has only two, especially if coital exposure is frequent and long term; and when you have multiple partners, you are not only putting yourself at risk, but you are also putting your loved ones at risk. This message appears to be a particularly important one and one that may help change the social norms around multiple partnering.

In any case, these messages appear rather indisputable in preventing an infection that is spread sexually, even if one may be skeptical about whether concurrency *per se *plays a vital role in that ongoing transmission.

## Conclusions

Earlier arguments for a key role of concurrent sexual partnerships in the genesis of generalized epidemics were primarily based on ethnographic and related sexual behavioural data and early modelling. The complex nature of the epidemics and the limitations of the available data made that evidence amenable to some understandable questioning. More recent and improved modelling, as well as better analysis of behavioural data, continues to support a key role of concurrency.

In addition, however, we have presented here a variety of epidemiologic evidence that has heretofore largely not been included in the discussion. Together, the modelling, the behavioural evidence, these multiple components of epidemiologic evidence and the lack of any other plausible explanation for these rapidly propagating epidemics make for an extremely compelling body of evidence. Moreover, specific messaging, bringing together the concepts of multiple partnering and concurrency, appears reasonable and salient in promoting safer individual behaviour, as well as positive social norms.

## Competing interests

The authors declare that they have no competing interests.

## Authors' contributions

TLM and JDS collaborated equally in writing the article. All authors read and approved the final manuscript. The views expressed here are not necessarily those of the US Agency for International Development (USAID).

## Authors' information

TLM is a Senior HIV Prevention Advisor in the Office of HIV/AIDS at USAID. JDS is the Science Advisor for the Bureau for Global Health at USAID.
